# The NJ Alliance for Clinical and Translational Science (NJ ACTS) experience: Responding at “warp speed” to COVID-19

**DOI:** 10.1017/cts.2022.383

**Published:** 2022-04-04

**Authors:** Pragya Thaman, Barbara Tafuto, Céline Gélinas, Sunanda Gaur, Judith A. Neubauer

**Affiliations:** 1 Ernest Mario School of Pharmacy, Rutgers University, Piscataway, NJ, USA; 2 Department of Health Informatics, Rutgers School of Health Professions, Newark, NJ, USA; 3 Center for Advanced Biotechnology and Medicine, and Department of Biochemistry and Molecular Biology, Rutgers Robert Wood Johnson Medical School, Piscataway, NJ, USA; 4 Department of Pediatrics, Clinical Research Center, Rutgers Robert Wood Johnson Medical School, New Brunswick, NJ, USA; 5 Department of Medicine, Division Pulmonary and Critical Care Medicine, Rutgers Robert Wood Johnson Medical School, New Brunswick, NJ, USA; 6 NJ Alliance for Clinical and Translational Science (NJ ACTS), New Brunswick, NJ, USA

**Keywords:** COVID-19, Institutional Review Board, CTSA, clinical research, regulatory

## Abstract

**Introduction:**

The COVID-19 pandemic’s need for life-saving treatments and a "warp speed" vaccine challenged the National Institutes of Health’s Clinical and Translational Science Award (CTSA) recipients to improve their methods and processes in conducting clinical research. While CTSA recipient, New Jersey Alliance for Clinical and Translational Science (NJ ACTS), responded to this call to action with significant clinical research milestones, a comprehensive understanding of regulatory metrics during the COVID-19 pandemic is uncertain. The objective of this research is to identify, compare, and contrast metrics that illustrate the effectiveness of NJ ACTS’s research mobilization efforts during COVID-19.

**Methods:**

Data were collected from the Institutional Review Board (IRB), the Clinical Research Units (CRUs), and the Office of Research and Sponsored Programs (ORSP). IRB data detailed the volume and types of protocols approved and turnaround time (TAT) for approval in 2020 vs. 2019. CRU data examined study metrics of adult and pediatric clinical trials across 2018-2020. ORSP data documented awards received in 2019 and 2020

**Results:**

Analysis revealed a 95% increase in IRB-approved studies in 2020, with a significant decrease in TAT for COVID-19 studies. All CRUs observed a median 5.2-fold increase in the enrollment of adult and pediatric participants for COVID-19-related research. Study income was 106% and 196% greater than 2019 and 2018, respectively, with more than half funded through federal sponsors and 89% for COVID-19 trials. ORSP data revealed that 9% of awards and 26% of 2020 funding were COVID-19 studies.

**Conclusion:**

This study demonstrates that NJACTS effectively responded to challenges posed by the pandemic

## Introduction

Bench-to-bedside translational research can take up to 17 years, hindering efficient and optimal healthcare outcomes [1]. Challenges to clinical and translational research include IRB turnaround time (TAT), study accrual, and funding. IRB reviews are seen as a time-consuming process, with TATs reported to be around two weeks to four months before receiving IRB approval in the best-case scenario [2]. Inability to meet subject accrual goals in clinical trials can often be inefficient and wasteful, limit generalizability of results, and lead to premature closure of trials [3].

While translating research from bench to bedside can be a challenge under normal circumstances, the restrictions on academic medical centers during the COVID-19 pandemic magnified that challenge. Faced with unanticipated deviations to protocols, clinical researcher remote work requirements, and restrictions on study enrollment, Clinical Research Units (CRUs) at the 60+ CTSA hubs were forced to evolve at a record pace in the wake of the unforeseen consequences of the COVID-19 pandemic [4]. The CRUs at Rutgers’ New Jersey Alliance for Clinical and Translational Science (NJ ACTS) were no exception when NJ Executive Order No. 104 limited on-site academic medical center research activities to only those related to developing COVID-19 testing, clinical trials associated with the treatment or prevention of COVID-19, trials considered to be life-saving, and other critical SARS-CoV-2-related research activities [5]. This not only resulted in a pause in enrollment into ongoing or new non-COVID-19 human subject research studies but was accompanied by an increased need to rapidly approve, start, and enroll subjects into urgently needed COVID-19 clinical research studies. Such a call to action put the value of the national CTSA program to the test and NJ ACTS responded in kind.

Five major cores of NJ ACTS played a significant role in implementing the internal infrastructure that streamlined the efficiency of the clinical trial process within Rutgers CRUs. The NJ ACTS Clinical Trials Office (CTO) in collaboration with its Regulatory Core and its Participant and Clinical Interactions (PCI) Core formed committees that vetted the feasibility of proposed studies and prioritized COVID-19 studies for enrollment. The cores developed procedures that improved virtual operations for informed consent and implemented other opportunities to decentralize study procedures where practical [6,7]. The NJ ACTS Community Engagement Core pivoted to virtual platforms to increase enrollment in NJ ACTS-specific research and was able to engage community and healthcare organization partners to co-design and implement successful COVID-19 research design and recruitment strategies. The NJ ACTS Workforce Development Core procured and provided training modules for new hires at the CTO and recruited graduate students from the clinical research management program to serve as volunteer clinical research associates working with the CRUs on COVID-19 trials in areas of consenting and data management.

As a result of NJ ACTS efforts, the Rutgers CRUs were able to enroll 840 participants in the Johnson & Johnson COVID-19 vaccine trial alone in just 6 weeks, one of the highest enrolling sites for that trial in the USA. What remained unclear was the effectiveness of the newly devised translational process for IRB approvals and patient accruals on all research throughout the pandemic lockdown at Rutgers CRUs.

This research evaluates the overall efficiency and effectiveness of the IRB approval process and of subject accrual at Rutgers for research activities during the 2020 COVID-19 lockdown which was facilitated through the activities of NJ ACTS. The Regulatory Core assessed the metrics of IRB TAT and subject accrual, among others, and determined the overall impact of NJ ACTS on the ability to respond effectively and efficiently to the unprecedented research needs of its institutions during the COVID-19 pandemic.

## Methods

Data were compiled from three research units at Rutgers: the Institutional Review Board (IRB), the Clinical Research Units (CRUs), and the Office of Research and Sponsored Programs (ORSP). The datasets were analyzed using descriptive analytics.

### Rutgers Institutional Review Board (IRB)

The first dataset was collected from the Rutgers IRB, which includes the Health Sciences-New Brunswick IRB, the Health Sciences-Newark IRB, and the Arts and Sciences IRB. Industry-sponsored clinical trials are reviewed by a commercial IRB and categorized as Western IRB (WIRB) or Facilitated Review. The Rutgers IRB uses a fully electronic IRB system (Huron eIRB) which has been implemented University-wide for several years. The eIRB platform allowed for a systematic and robust analysis of the IRB processes. For the purposes of this study, median TATs in calendar days, from protocol submission to approval, were requested for protocols approved by the IRB in 2019 and 2020. The data were divided into four categories: COVID-19-related protocols originally approved in 2019 (see below), non-COVID-19-related protocols approved in 2019, COVID-19-related protocols approved in 2020, and non-COVID-19-related protocols approved in 2020. The data were then further analyzed by review type (Exempt, Expedited, Full Board IRB Review, Non-Human Subject Determination, and WIRB). Additionally, TATs were analyzed based on principal investigator (PI) response time, IRB reviewer time, and IRB office processing time, or total IRB review TAT. At the beginning of the pandemic, studies were qualified as COVID-19-related if they mentioned “COVID-19,” “SARS-CoV-2,” or “Coronavirus” in the title. At the end of June 2020, the eIRB application itself required applicants to identify if their study was COVID-19-related. Some non-COVID-19 studies that were submitted for review in 2019 were modified into COVID-19-related studies after the start of the pandemic and are thus classified as COVID-19 studies approved in 2019 by the IRB.

### Rutgers Clinical Research Units (CRUs)

The second aim of our study was to compile clinical trials data from the Rutgers CRUs within the Rutgers Biomedical Health Sciences (RBHS). RBHS has five CRUs: New Brunswick Adult CRU, New Brunswick Pediatric CRU, Newark CRU, Environmental and Occupational Health Sciences Institute (EOHSI) CRU, and School of Dental Medicine CRU. The first three have the largest volume of clinical studies, while the other two CRUs (EOHSI and School of Dental Medicine) have small portfolios of clinical research studies which focus on areas of environmental and oral health, respectively.

Specifically, the dataset focused on collecting and analyzing clinical trials from both the adult and pediatric populations conducted between the years 2018-2020. These studies were identified as adult or pediatric, and total enrollment and study income was computed by year for each year of active study enrollment. In addition, studies were identified as being COVID-19-related using the terms “COVID-19,” “SARS-CoV-2,” or “Coronavirus” in the title of the study. Each CRU study enrollment and study income was grouped by year and classified as COVID-19 vs. non-COVID-19 trials for adult population and pediatric population studies.

### Rutgers Office of Research and Sponsored Programs (ORSP)

The last dataset was collected from the Rutgers Office of Research and Sponsored Programs (ORSP). The Rutgers ORSP oversees funding for studies conducted at Rutgers Camden, Rutgers Newark, Rutgers New Brunswick, and Rutgers Biomedical and Health Sciences. Data collected from the ORSP included the number of awards received and total costs in the initial period between 2019 and 2020. The 2020 data were further subdivided into COVID-19-related studies and non-COVID-19 studies.

## Results

### Comparisons of IRB-Approved Protocols in 2019 and 2020, Between non-COVID-19 and COVID-19-Related Studies

Table 1 shows the total number of protocols approved during 2019 and 2020 (non-COVID-19 and COVID-19), and the time to approval by review category (Exempt, Expedited, Full Board, Non-Human Determination, and Western IRB). The overall number of new IRB protocols submitted for approval increased from 1,248 in 2019 to 2,433 in 2020 (reflecting a 95% increase in the volume of protocols in 2020 vs. 2019). The 2,433 protocols in 2020 included 406 COVID-19-related clinical research studies, which was largely the only type of clinical research that was allowed to move forward with study implementation and recruitment during the University’s restrictions due to the pandemic. There was also an increase in the number of exempt, expedited, and full board review non-COVID-19-related protocols during this period. The largest absolute increase was in the number of expedited protocols (924 vs. 300; a 208% increase vs. 2019), and full board review protocols (112 vs. 32; a 250% increase over 2019), whereas exempt non-COVID-19 protocols (661 vs. 556) increased 16% compared to 2019.

**Table 1.** Total number of non-COVID-19 and COVID-19 protocols approved in 2019 and 2020 and the time to approval by review category

	2019	2020 non-COVID-19	2020 COVID-19
**Total # Protocols Approved**	1,248	2,027	406
**Time to Approval (Days)**			
**Non-Human Research Determination**	11 (7, 24) ^†^	12 (5, 25)	5 (1,12)
**Exempt**	30 (21, 71)	27 (14, 50)	13 (6, 23)
**Expedited**	59 (38, 124)	38 (17, 69)	14 (6, 36)
**Full Board IRB**	119 (48, 176)	80 (50, 136)	23 (13, 53)
**WIRB**	35 (29, 47)	28 (18, 41)	9 (8, 12)

^†^Median (Lower Quartile, Upper Quartile).

IRB: Institutional Review Board; WIRB: Western Institutional Review Board.

In 2019, the median times to IRB approval were dependent upon the type of protocol, with non-human research determinations taking the least amount of time, and the time to approval increasing from exempt, to expedited, to full board IRB approvals (Table 1). The value of contracting with a commercial IRB (e.g., WIRB) for industry-sponsored clinical trials was noteworthy since the WIRB approval times for industry-sponsored clinical trials were much shorter than the time to approval for investigator-initiated protocols reviewed by the Rutgers IRB.

Also shown in Table 1 is that during 2020, the median times to approval for non-COVID-19-related studies in 2020 were similar to those in 2019, although there was a tendency toward faster median approval times for every type of protocol review, particularly for expedited and full board studies. Thus, even with the very significant increase in the volume of IRB protocols submitted during the pandemic, the review and administrative processes were efficient and not overwhelmed by the increase in volume of protocols during the pandemic shutdown. Of particular interest, Table 1 also shows that the median times to approval for COVID-19-related studies were significantly shorter for all protocol types compared to non-COVID-19 protocols approved in 2019 and 2020. This suggests that, in addition to the greater efficiency of the IRB approval process observed in 2020, there was additional effort and attention to assuring that the COVID-19-related protocols were approved as quickly as possible.

**Fig. 1.**
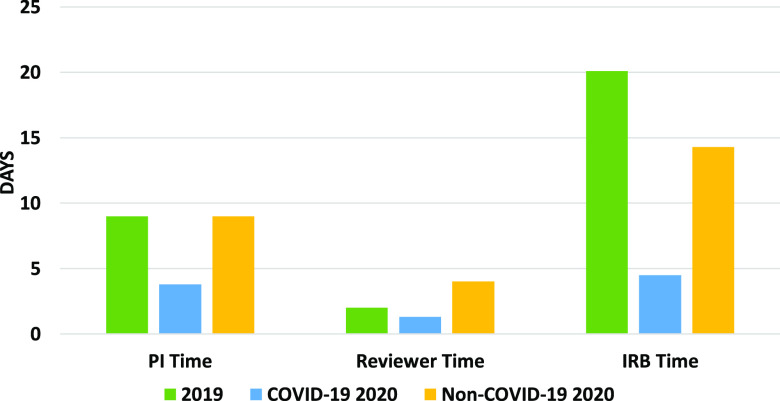
Proportion of time during the Rutgers IRB protocol approval process associated with the study PI, the IRB reviewer, and IRB staff for IRB protocols approved in 2019 and 2020 for non-COVID-19 and COVID-19-related protocols. This excludes WIRB studies. PI: Principal Investigator; IRB: Institutional Review Board.

Further analysis of the proportion of time in the Rutgers IRB approval process associated with the study PI, the reviewer, and the IRB showed that the time taken at every point of contact with the protocol was shortened during the approval process for COVID-19 studies in 2020 (Fig. 1). Analyzing the TATs based on the different stages of this approval process, we found that studies approved in 2019 took an average of 9.0 days for PI response, 2.0 days for reviewer time, and 20.1 days for IRB staff review. For COVID-19 studies approved in 2020, these TATs were 3.8 days for PI response, 1.3 days for reviewer time, and 4.5 days for IRB review time. Lastly, for non-COVID-19 studies approved in 2020, the TATs were 9.0 days for PI review, 4.0 days for reviewer time, and 14.3 days for IRB review. Overall, these data reveal that the significantly decreased TATs for 2020 COVID-19 studies can be credited to the collective efforts of the PIs, IRB reviewers, and the IRB staff. The maintenance of the TATs for non-COVID-19 studies in 2020 in the face of the significantly increased protocol volume compared to 2019 was largely due to a shorter TAT at the IRB staff level.

**Fig. 2.**
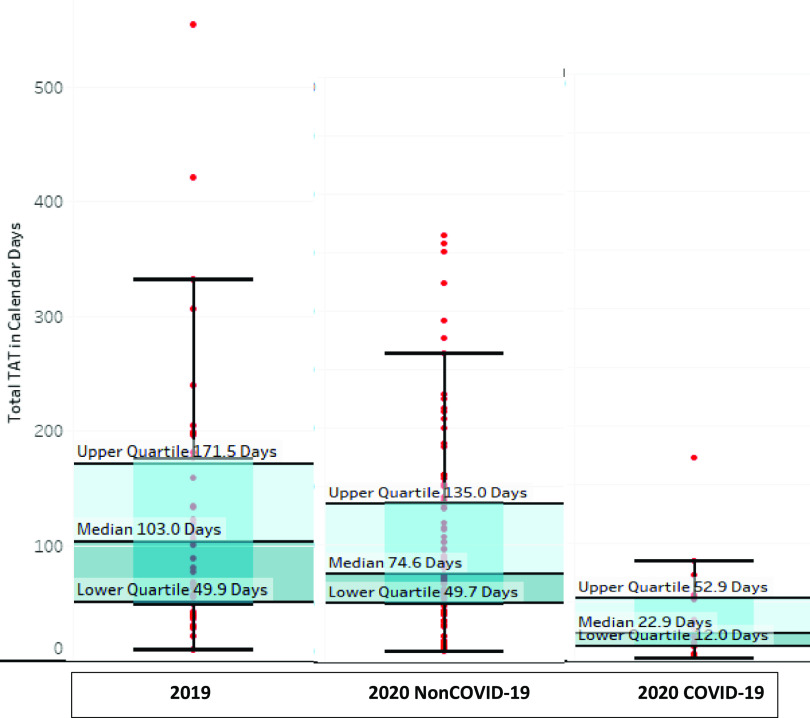
Whisker plots of the total time to approval for full board IRB protocols reviewed by the Rutgers IRB and approved in 2019 and for non-COVID-19 protocols and COVID-19-related protocols approved in 2020. This excludes WIRB studies. Note that the median time to approval declined in 2020 compared to 2019 for both non-COVID-19 and COVID-19-related protocols with the shortest time to approval for the 2020 COVID-19 protocols. Additionally, observe that the reduction in time of the outliers and the upper and lower quartile values in 2020 compared to 2019. IRB: Institutional Review Board; TAT: Turnaround time to IRB approval.

The increased efficiency in the time to approve Rutgers IRB protocols in 2020 is further evidenced by the tightening of the variance in the approval times. This is demonstrated in Fig. 2 which shows the whisker plots for the full board IRB approvals, which is the category of review that generally takes the longest time. Of note in Fig. 2 is that the median, upper quartile, lower quartile, and outliers are all reduced in the 2020 full board reviews compared to the 2019 full board reviews, with the shortest times seen for the 2020 COVID-19-related IRB projects.

**Fig. 3.**
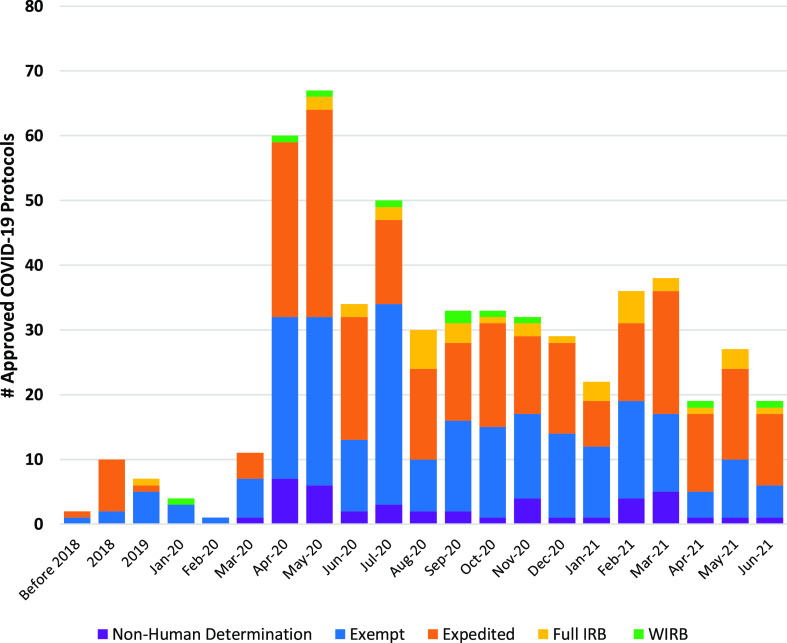
Volume of COVID-19 protocols by month and review type. The number of approved COVID-19-related IRB protocols increased abruptly after the March 16^th^ COVID-19 Emergency Executive Order with a sustained increase over the next 15 months. Note that there were several modifications to previously approved IRB protocols that were submitted to include a focus on COVID-19. IRB: Institutional Review Board; WIRB: Western Institutional Review Board.

It is noteworthy that there was a rapid increase in the number of COVID-19-related research protocols submitted to the IRB immediately after the emergency shutdown in mid-March 2020, which has been sustained throughout the 15 months covered by this review (Fig. 3). In addition, there were several previously approved research protocols in which the investigators pivoted their research and submitted modifications to focus on COVID-19. This included seven protocols approved in 2019 and an additional 13 protocols approved prior to 2019.

### Comparisons of Subjects Enrollment and Study Income for Clinical Research Studies conducted in the Clinical Research Units and Opened in 2019 Compared to 2020 (non-COVID-19 and COVID-19-Related Studies)

Although enrollment of new subjects into ongoing “non-life-saving” studies or new non-COVID-19 human subject research was abruptly paused following the NJ Governor’s edict in March 2020 due to the pandemic, there was a pressing need to rapidly enroll subjects into approved COVID-19 clinical research studies. The Rutgers CRUs rose to this unprecedented challenge and the urgent call to action to conduct critical clinical trials to identify life-saving treatments and vaccines against COVID-19, enrolling diverse participants from the community and throughout New Jersey, including minority communities who have been disproportionately affected by the pandemic. These included three large studies: a Cohort study of SARS-CoV-2 incidence, transmission, and disease severity in healthcare workers [8,9], a Phase 3 trial to evaluate the safety and efficacy of the Johnson & Johnson COVID-19 vaccine [10], and a Phase 3 trial to evaluate the safety, efficacy, and immunogenicity of the Moderna COVID-19 vaccine [11]. Other studies included trials examining an array of clinical interventions on patients hospitalized with COVID-19 such as remdesivir [12], methodological studies for detecting SARS-CoV-2, or studies assessing methods to purify air of the virus (EOSHI CRU). NJ ACTS played a pivotal role in the implementation of the necessary infrastructure and processes to enable the rapid approval, start-up, and execution of these studies by the CRUs. The NJ ACTS CTO and its Regulatory and PIC Cores were particularly instrumental in the development of quality study protocols and assuring feasibility, facilitating a streamlined and efficient study approval process. In addition to committees prioritizing studies for enrollment, the Workforce Development Core provided training modules for new hires at the CTO. The Workforce Development Core also allowed the CRUs to mobilize and rapidly expand their study personnel to meet the demands associated with the unprecedented scale and “warp speed” timeline for the enrollment of subjects, and full implementation of the J&J vaccine trial, through the recruitment of graduate students from the clinical research management program to serve as volunteer clinical research associates to help with consenting and data management.

Of interest is that, although the mix of actively enrolling studies shifted from non-COVID-19 studies to COVID-19 studies, the total number of clinical studies conducted in the CRUs did not significantly change during 2020 (n = 114) compared to 2018 (n = 116), and 2019 (n = 140).

**Fig. 4.**
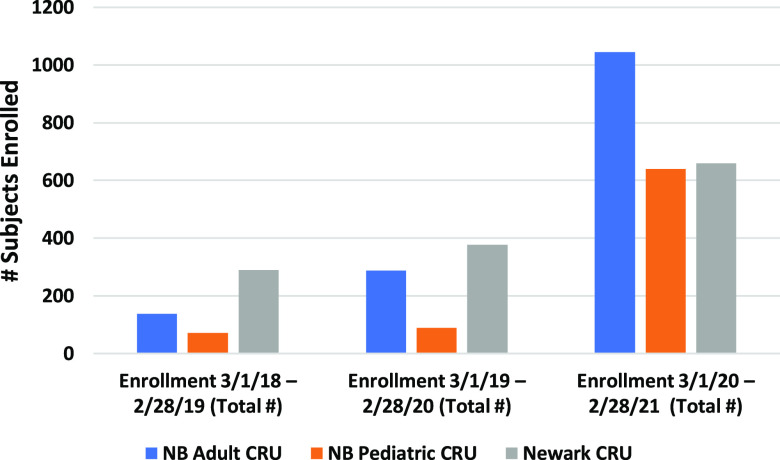
Clinical Research Units subject enrollment for the period from March 1 to February 28 for the years 2018, 2019, and 2020. Note that while enrollment in non-COVID-19-related studies was paused in 2020, there was an increase in subject enrollment for all three of the major CRUs. NB: New Brunswick; CRU: Clinical Research Unit.

The extraordinary challenge to meet the goals of the COVID-19 studies was met with a degree of efficiency that resulted in a significant increase in the enrollment of participants into COVID-19-related research at all CRUs, with a median 5.2-fold increase (range 1.8-9.0-fold) between March 1, 2020, and February 28, 2021, compared to similar time periods in 2018-2019 and 2019-2020 (Fig. 4). Of note, increased enrollment was observed in both the adult and pediatric CRUs. While smaller in the number of studies and enrollment volume, the School of Dental Medicine and EOHSI CRUs also participated by increasing their focus on COVID-19-related studies.

Consistent with the increased subject enrollment, there was also a significant increase in clinical study income. Total study income for the CRUs between March 1, 2020, and February 28, 2021, was $8,582,564 compared to a similar time periods in 2018-2019 and 2019-2020 in which study income was $2,895,421 and $4,146,856, respectively. In total, Rutgers’ efforts to meet the challenges of the SARS-CoV-2 pandemic through clinical, translational, basic, behavioral, social science, and global health initiatives contributed 26% of all award funding received in 2020 (data not shown).

## Discussion

The results show how NJ ACTS rose to the challenge and spearheaded efforts to prioritize and accelerate the review and approval of COVID-19 studies, study activation, and subject enrollment, and supported research efforts that translated into increased funding to advance critically needed research. Specifically, our analyses showed that 1) although there was a 2-fold increase in the number of IRB protocols submitted in 2020 compared to 2019, the time to approval was significantly shorter for COVID-19-related studies without any detriment to the approval times for non-COVID-19 protocols, and 2) while non-COVID-19 clinical trials were largely closed to enrollment, the CRU enrollment of subjects into critical COVID-19-related trials was robust and significantly exceeded pre-COVID-19 pandemic subject accrual rates. Overall, these results support the notion that the NJ ACTS infrastructure successfully interfaced with key institutional units to rapidly implement COVID-19 clinical trials by establishing innovative policies and procedures to improve efficiencies essential to the clinical trial process.

Past studies which analyzed multisite protocols from multiple IRBs describe IRB review times ranging from 13 to 116 days, where 220 days was the average range between the shortest and longest time for approval [2]. This report documents the “warp speed” response exhibited by NJ ACTS during the COVID-19 pandemic. Our analysis revealed that in 2019, the Rutgers IRB exhibited average review times within the aforementioned range. TATs to approval improved significantly for COVID-19 studies during the pandemic. Improvement of TAT was observed across the board for all types of review in 2020. The most dramatic improvement in overall TAT was seen with studies undergoing full board review (80% improvement), followed by those undergoing expedited review and WIRB studies. This notable improvement in IRB approval times suggests a mobilization of efforts supported by NJ ACTS. This is in alignment with a recent report showing that across all CTSA institutions surveyed 52% of COVID-19 protocols were reviewed much faster and 41% somewhat faster than other protocols [13].

Despite the prioritization of COVID-19 studies, Rutgers IRB review times for non-COVID-19 protocols in 2020 were not negatively affected by the dramatic surge in the number of protocols submitted for IRB review. In fact, TAT to approval for most types of non-COVID-19 protocols also generally improved in 2020 compared to 2019 studies which were approved prior to the pandemic. Non-COVID-19 2020 protocols undergoing full board or expedited review saw more improvement in TAT compared to those protocols approved in 2019. Non-COVID-19 2020 WIRB protocols also showed an improvement over their 2019 counterpart. This is particularly noteworthy since the total number of protocols submitted to the Rutgers IRB skyrocketed during this time, with a 95% increase in the volume of protocols being approved. The 62% increase in new non-COVID-19-related IRB protocols in 2020 compared to 2019 suggests that the curtailed on-site laboratory and general clinical research provided clinical investigators an opportunity to prepare IRB protocols as well as, presumably, manuscripts and grant applications. Even though there was a high priority placed on approving COVID-19-related protocols, this prioritization was not to the detriment of the non-COVID-19 protocols which also benefited from the process efficiencies put in place at Rutgers during the pandemic. This reflects an overall improvement of the IRB review process to adapt to the challenges of the pandemic. This included the creation of a “COVID rapid response team” of senior IRB members, composed of both faculty and IRB staff, to meet the demand to review the influx of studies. During the surge, this required some extended working hours on the part of some IRB staff on this team to meet the demand. Additionally, the prior implementation of an entirely electronic IRB process at Rutgers was a critical factor that helped to expedite the review process remotely.

An in-depth examination of the results found that the time spent at each stage of the Rutgers IRB review process during the pandemic yielded a decrease in TAT on the part of the investigators, the reviewers, and the IRB office. The decrease in TAT for the IRB office was especially impressive for both COVID-19-related protocols, as well as for non-COVID-19 protocols during this period, in the face of an increase in workload and greater logistical challenges. Our analysis highlighted the collective effort displayed by Rutgers’ investigators, reviewers, and the IRB staff, who all contributed to the accelerated approval times. While it has been suggested that most of the increase in review capacity across the CTSA institutions resulted from additional efforts by IRB staff and members [13], the experience at Rutgers suggests that a collective effort on the part of the investigators, the reviewers, and the IRB staff could lead to sustained improvements going forward. Indeed, the success of the COVID-19 rapid response team approach at Rutgers has led to the permanent implementation of a “Pre-Review Process” where the IRB now provides courtesy review of submissions prior to IRB review, as well as pre-review of submissions that were tabled at a full board meeting prior to resubmission, as well as an optional service for studies approved with subcommittee review.

As noted in recent reports, efforts to expedite study start-up need to focus not only on the IRB approval process but also on the other requirements for study start-up and activation [6,13]. One of these requirements that can often delay study start-up is completion of clinical trial contract negotiation and execution. Fortunately, NJ ACTS implemented a streamlined process for the review of all non-oncology clinical trial contracts through the RBHS CTO just prior to the pandemic, which helped to prioritize and fast-track the review of COVID-19 studies. The availability of an electronic system for submission of all grants and contracts greatly facilitated a smooth and efficient transition to virtual operation during the pandemic. Going forward, this structure should also help other non-COVID-19 studies navigate contract negotiation and budget review in a more efficient way.

A barrier frequently encountered by researchers conducting clinical and translational research is often the limited number of clinical trial participants. It is in fact one of the hurdles addressed by the NIH’s CTSA RFA, which highlights the importance of recruiting diverse pools of research participants from the local community in order to address the need for study subjects, as well as inclusion of minority populations representative of the community. Successful CTSA applicants place great importance in interacting with the local community through various initiatives to address this need. Consistent with this, the Common Metrics Workgroup identified “studies meeting accrual goals” and “notice of grant award to first accrual” as essential common metrics for evaluating the efficiency of research and success in achieving CTSA goals [14]. In our analysis, we found a sharp increase in subject enrollment in 2020, during the time of the pandemic, with a median 5.2-fold increase as compared to 2019 and 2018 studies. A significant increase was also observed in the pediatric population. Additionally, we observed a 61% decrease in median time for subject accrual from the time the study first became activated (data not shown). Together, these results reflect the robust and comprehensive recruitment efforts put in place in response to the urgency of the pandemic, the vast number of potential participants, and the inclusion of vulnerable populations from the community. The processes implemented by the multiple NJ ACTS cores to recruit subjects from the community will be invaluable to build upon subject enrollment diversity to further engage the NJ population in future clinical trials. An additional level of efficiency was achieved when the Rutgers CRUs put in place a committee of physicians and scientists to prioritize COVID-19 studies for enrollment of subjects in the intensive care units (ICUs) and COVID-19 inpatient units. Furthermore, the CTO created a “Vetting Committee,” with representation from the Newark CRU and the New Brunswick CRU and from the RWJ Barnabas Health System, to evaluate every COVID-19 study request from sponsors to determine feasibility within Rutgers at both CRUs. While these mechanisms were developed to help optimize the conduct of COVID-19 studies, these initiatives can be implemented to have a broader and lasting impact to improve the management of all clinical studies across Rutgers going forward.

In addition to limited numbers of clinical trial participants, CTSA institutions have reported barriers to research due to immense research costs and a lack of funding sources to support it. This hinders the development of breakthrough therapies and consequently their application for public benefit. The Rutgers CRUs, however, reported that studies received the greatest amount of funding seen in the past few years with a 106% and 196% increase in clinical trials income in 2020 versus 2019 and 2018 (data not shown). The substantial rise in study income, with over half being funded through federal partnerships, shows a clear interest in advancing the scope of research and accelerating progress on these important studies. In some instances, the majority of funding was dedicated to COVID-19 trials, emphasizing the urgency and need for discoveries to be made to contain the pandemic.

Altogether, the metrics analyzed in this study to assess the efficiency and efficacy of the NJ ACTS’ “warp speed” response validate its undeniable value as a CTSA hub. The metrics show that NJ ACTS rose to the challenges posed by this pandemic and fulfilled its responsibilities as a CTSA recipient, by rapidly making notable process improvements to facilitate progress in clinical and translational research during this crisis. Importantly, there have been challenges and lessons learned that have provided unique opportunities for new processes to be put in place, many of which can lead to sustained improvements to benefit all studies going forward [6,7].
